# Principles and development of collagen-mediated tissue fusion induced by laser irradiation

**DOI:** 10.1038/s41598-019-45486-4

**Published:** 2019-06-28

**Authors:** Shun Sasaki, Tetsuo Ikeda, Shin-ichiro Okihara, Shotaro Nishimura, Ryu Nakadate, Hiroshi Saeki, Eiji Oki, Masaki Mori, Makoto Hashizume, Yoshihiko Maehara

**Affiliations:** 1 Department of Surgery and Science, Graduate School of Medical Sciences, Kyushu University, Fukuoka, Japan; 2 Endoscopy and Endoscopic Surgery, Fukuoka Dental College, Fukuoka, Japan; 3 Graduate School for the Creation of the New Photonics Industries, Shizuoka, Japan; 4 Graduate School of Bioresource and Bioenvironmental Sciences, Faculty of Agriculture, Kyushu University, Fukuoka, Japan; 5 Department of Advanced Medical Initiatives, Graduate School of Medical Sciences, Kyushu University, Fukuoka, Japan; 6 Department of Surgery, Kyushu Central Hospital, Fukuoka, Japan

**Keywords:** Tissues, Medical research

## Abstract

The mechanism underlying tissue fusion mediated by laser irradiation remains unclear. We clarify the mechanisms underlying laser-mediated tissue fusion using a novel model. Microscopic examinations of morphological changes within the adventitia of a bovine carotid artery and a collagen sheet prepared from bovine dermis showed collagen fibril bundle loosening and collagen fibre swelling following heating at 46 °C. An incised bovine carotid artery covered with a collagen sheet to which pressure and laser heat of 40 °C–52 °C were applied created a structure that was pressure resistant to >300 mmHg. Microscopic analyses of the irradiation site showed collagen fibril interdigitation. Hence, low-temperature laser-mediated tissue fusion causes collagen fibril bundles to loosen and swell, and crimping causes the fibres to intertwine. As the temperature declines, the loosened and swollen fibrils and fibres tighten, and collagen fibre interdigitation is completed. This technology could be applied to fuse tissues during surgery.

## Introduction

Currently, the main surgical techniques for tissue fusion are mechanical methods, such as suturing with needle and thread, stapled and compression anastomosis, and dehydration fixation (mylarisation), such as the use of high frequency electrosurgery equipment and ultrasonic dissection coagulation apparatus^1–3^. Unfortunately, these procedures have various drawbacks that can interfere with wound healing, including tissue damage due to puncture, impeded blood flow due to excessive compression, and irreversible protein denaturation by heat injury. In recent years, function preservation has been regarded as an important feature of surgical procedures, leading to the demand for the development of technologies that can fuse tissues whilst leaving them in a viable state.

Collagen, which comprises fibrous proteins within the extracellular matrix, accounts for approximately 30% of the body’s protein, and it is abundant in the skin, bones, tendons, blood vessels, and teeth^4–6^. The adventitia or outermost layer of the arterial wall comprises mainly of type 1 collagen^7,8^. Three collagen molecules with a repeating Gly-X-Y amino acid sequence form the triple helical structure of tropocollagen (diameter: 1.5 nm) that is crosslinked, which gives the resulting fibrils a striped appearance. These collagen fibrils are bundled together to form fibres (diameter: 1–20 µm) that maintain an organ’s shape^9–12^. When the temperature of the collagen exceeds body temperature, its structure can change^13^. Heating collagen with a laser swells collagen fibrils^14^. Laser irradiation successfully fused tissues in blood vessels, nerves, lungs, and other organs^15–19^, and photochemical tissue bonding with laser has been reported to be a less damaging sutureless technique for tissue fusion^20–22^. However, the basic processes underlying tissue fusion remain unclear. We clarify the morphological changes of collagen that occur in response to low-temperature heating. Further, we established a novel tissue fusion method, which allows contact-free local heating and retains tissue viability with very little damage, and developed an understanding of the collagen-related processes that underpin laser-induced tissue fusion.

## Results

### Heat-induced morphological changes to biological artery tissue and a biomaterial-derived collagen sheet

**Figure 1:**
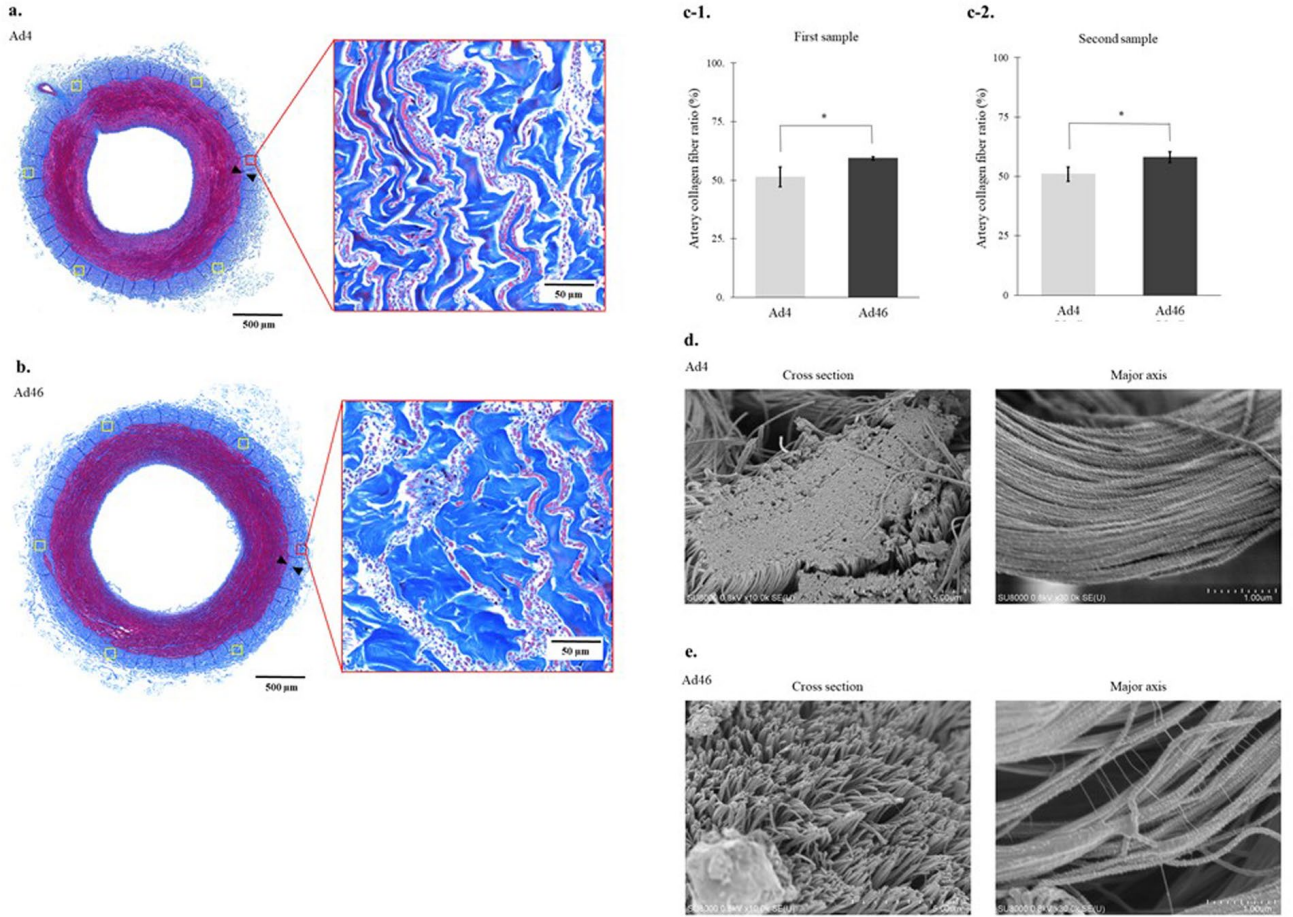
Heat-induced morphological changes to the collagen fibres in the adventitia. (**a**) Optical microscopy shows that the collagen fibres in the arterial adventitia washed at 4 °C (Ad4) are present as dense overlapping layers of blue fibres with a collagen fibre area ratio of 47.8% (Masson’s trichome stain). (**b**) Optical microscopy shows that the collagen fibres are enlarged in the arterial adventitia washed at 46 °C (Ad46) with a collagen fibre area ratio of 60.5% (Masson’s trichome stain). **(c-1)** The mean collagen fibre area ratio in Ad46 (mean collagen fibre area = 59.4%, 95% CI = 58.6%–60.2%) is larger than that in Ad4 (mean collagen fibre area = 51.4%, 95% CI = 46.5%–56.3%) (P < 0.01, Wilcoxon’s nonparametric test). (**c-2**) The mean collagen fibre area ratio in Ad46 (mean collagen fibre area = 50.9%, 95% CI = 47.5%–54.4%) is larger than that in Ad4 (mean collagen fibre area = 58.0%, 95% CI = 55.4%–60.7%) (P < 0.01, Wilcoxon’s nonparametric test). (**d**) Scanning electron microscopy shows that, in cross section (×10,000) and along the major axis (×30,000), there are no gaps between the adventitial collagen fibrils in Ad4. **(e)**. Scanning electron microscopy shows that, in cross section (×10,000) and along the major axis (×30,000), the bonds between the collagen fibrils have loosened in Ad46 and have a filamentous fibrous structure, and collagen fibre swelling is revealed. The banded appearance of the collagen fibrils is evident. *Indicates statistical significance (P < 0.01).

**Figure 2:**
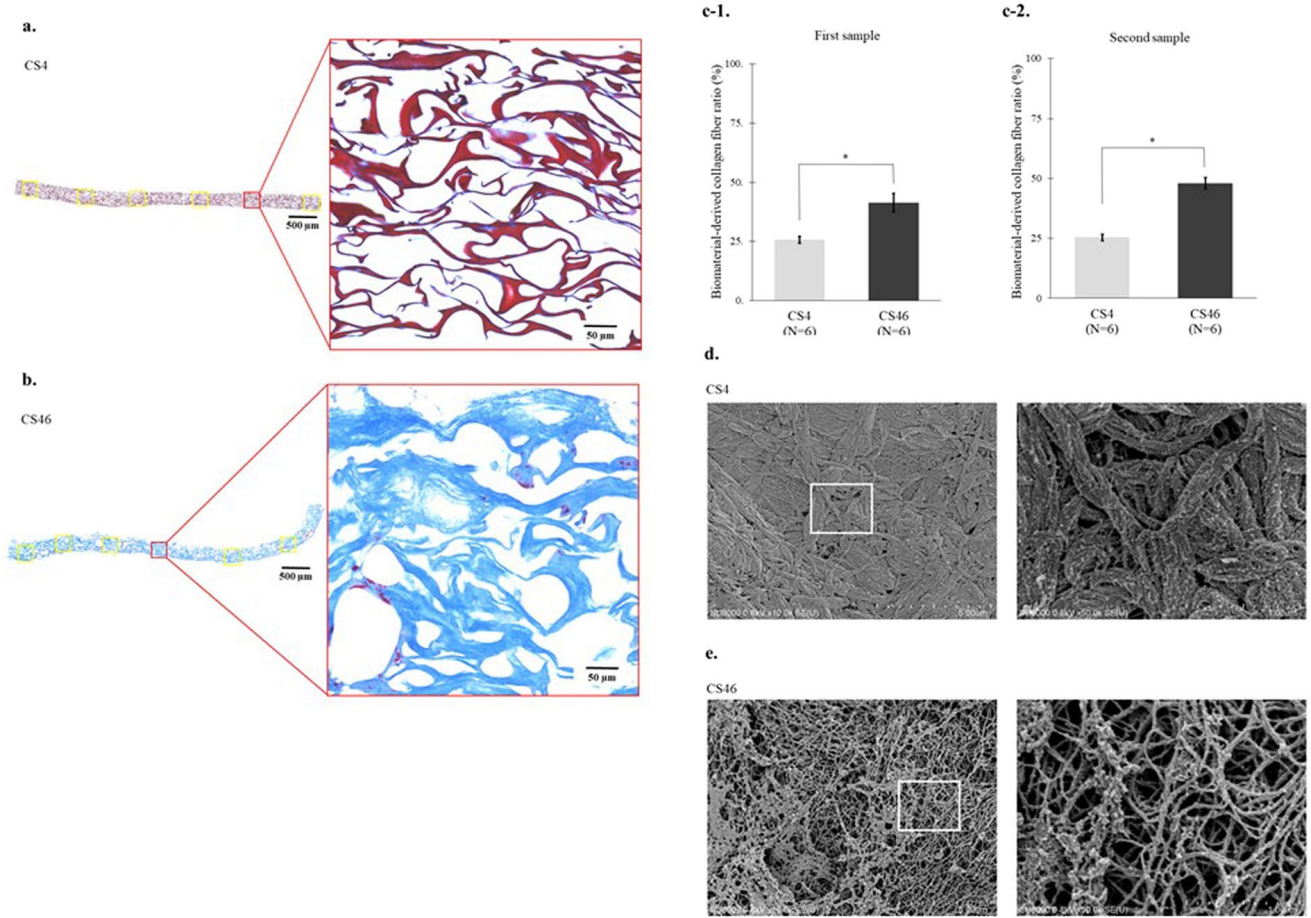
Heat-induced morphological changes to the collagen fibres in the collagen sheet. **(a)** Optical microscopy shows that the collagen sheet rinsed at 4 °C (CS4) has a mesh-like structure with multiple overlapping collagen fibres that stained red, but within the collagen fibres, the fibrous nature of the collagen fibrils is not evident (collagen fibre area ratio: 25.3%) (Masson’s trichome stain). **(b)** Optical microscopy shows that the collagen sheet rinsed at 46 °C (CS46) contains swollen collagen fibres that stained blue, and that the fibrous structure of the collagen fibrils within the collagen fibres is evident (collagen fibre area ratio: 43.9%) (Masson’s trichome stain). **(c-1)** The mean proportion of collagen fibres is significantly larger in CS46 (mean proportion = 41.4%, 95% CI = 36.8%–45.9%) than in CS4 (mean proportion = 25.7%, 95% CI = 24.0%–27.5%) (P < 0.01, Wilcoxon’s nonparametric test). (c-2) The mean proportion of collagen fibres is significantly larger in CS46 (mean proportion = 48.0%, 95% CI = 46.0%–49.9%) than in CS4 (mean proportion = 25.4%, 95% CI = 23.4%–27.3%) (P < 0.01, Wilcoxon’s nonparametric test). (**d**) Scanning electron microscopy shows CS4 collagen fibres, which densely overlaps to form a sheet without any gaps (×10,000), formed by collagen fibrils without a striped appearance (×50,000). (**e**) Scanning electron microscopy shows sponge-like CS46 with conspicuous gaps (×10,000), which consists of collagen fibrils that did not have a striped appearance and forms a polygonal mesh-like structure with individual fibrils at the edges (×50,000). *Indicates statistical significance (P < 0.01).

In the optical microscope low magnification (×10) findings, the adventitia washed at 4 °C (Ad4) and the adventitia washed at 46 °C (Ad46) were also observed as layers of blue collagen fibres (Fig. 1a,b). However, in the optical microscope high magnification (×200) findings, comparing the proportion of blue stained collagen fibres on the outside of the outer elastic plate, Ad4 vs. Ad46 is 51.4% (95% confidence interval (CI) = 46.5%–56.3%) vs. 59.4% (95% CI = 58.6%–60.2%) for the first sample (Fig. 1c-1) and 50.9% (95% CI = 47.5%–54.4%) vs. 58.0% (95% CI = 55.4%–60.7%) for the second sample (Fig. 1c-2); Ad46 in both samples showed a significantly higher occupancy rate (P < 0.01).

Figure 1d,e show scanning electron micrographs of the heat-induced morphological changes to the biological artery tissue. Scanning electron microscopy showed that in cross section (Fig. 1d) and along the major axis (Fig. 1d), there were no gaps between the adventitial collagen fibrils (mean diameter: 97.3 nm, 95% CI = 92.9–101.8 nm) in the Ad4 samples. In the Ad46 samples, the bonds between the collagen fibrils viewed in cross section (mean diameter: 123.6 nm, 95% CI = 119.2–128.1 nm) appeared looser, the fibrils exhibited a filamentous fibrous structure, and the collagen fibres appeared to be swollen (Fig. 1e). Similar morphological changes were evident when the samples were examined along the major axis, and the collagen fibrils exhibited a striped appearance (Fig. 1e). Thus, low-temperature heating loosened the bundled collagen fibrils and swelled the collagen fibres in the arterial adventitia. The same findings were confirmed whilst observing the second samples in each group.

The findings from the analysis of heat-induced morphological changes to the biomaterial-derived collagen sheet using the optical microscope are shown in Fig. 2a,b, and the relative proportions of collagen fibres within areas of the same size (484 × 484 µm) are shown in Fig. 2c. Optical microscopy showed that at a low magnification (×10), the samples of the collagen sheet that had been rinsed at 4 °C (CS4) had a mesh-like structure with multiple overlapping collagen fibres that stained red (Fig. 2a). At a higher magnification (×200), the structure of the collagen fibrils within the collagen fibres could not be confirmed (Fig. 2a). At a low magnification, the collagen fibres in the collagen sheet samples that had been rinsed at 46 °C (CS46) were swollen and stained blue, and at a higher magnification (×200), gaps between the collagen fibrils within the collagen fibres were evident when they were viewed in cross section (Fig. 2b). Compared with the CS4 samples (mean: 25.7%, 95% CI = 24.0%–27.5%), significantly higher proportions of collagen fibres were observed in the first CS46 samples (mean: 41.4%, 95% CI = 36.8%–45.9%) (P < 0.01) (Fig. 2c-1). The same finding was observed in the second sample (CS4 vs. CS46; 25.4% [95% CI = 23.4%–27.3%] vs. 48.0% [95% CI = 46.0%–49.9%], P < 0.01) (Fig. 2c-2). Scanning electron microscopy showed that the CS4 samples contained fine collagen fibrils that converged densely to form collagen fibre bundles (Fig. 2d), and at a higher magnification (× 50,000), striped structures were not evident within the biomaterial-derived collagen fibrils (mean diameter: 26.7 nm, 95% CI = 24.2–29.2 nm) (Fig. 2d). At a magnification of ×10,000, the morphology of the CS46 samples was sponge-like with conspicuous gaps (Fig. 2e), and at a higher magnification (×50,000), a striped appearance was not evident amongst the collagen fibrils (mean diameter: 26.9 nm, 95% CI = 24.4–29.4 nm); the collagen sheet exhibited a polygonal mesh-like structure with individual collagen fibrils at the edges (Fig. 2e). Low-temperature heating appeared to loosen the collagen fibrils and swell the collagen fibres in the collagen sheet. The same findings were confirmed whilst observing the second samples in each group.

### Fusion of collagen tissue using laser irradiation

#### Pressure resistance

**Figure 3:**
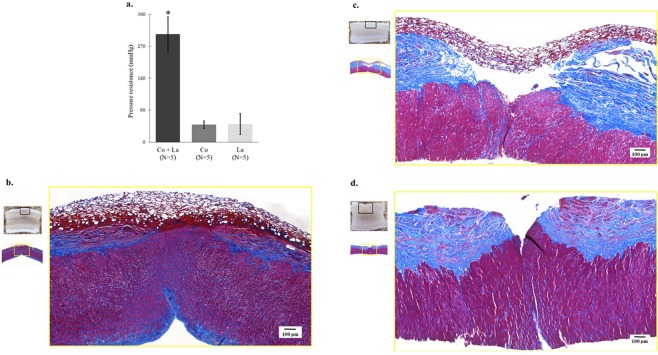
(**a**) Pressure resistance test results [Co + La vs. Co vs. La = 303.6 mmHg (95% CI = 353.0–254.2) vs. 50.8 mmHg (95% CI = 38.4–59.6) vs. 49.0 mmHg (95% CI = 21.3–80.3)]. Co + La group that underwent the entire laser tissue fusion procedure had a significant increase in the pressure resistance (P < 0.001, Wilcoxon’s nonparametric test). Co denotes the group that underwent collagen sheet and Teflon sheathing fusion without laser irradiation, whereas La denotes the group that underwent Teflon sheathing and laser irradiation without collagen sheet fusion. *Indicates statistical significance (P < 0.001). (**b**) Optical microscopy of Co + La group reveals that the incision causes the adventitia to contract at both ends, the adventitia is defective at the incision site. The adhesion site shows the mixed structure comprising the red fibres of the collagen sheet and blue fibres of the adventitia is confirmed (Masson’s trichome stain). (**c**) Optical microscopy of Co group shows a gap between the collagen sheets and the adventitia on one side, and a clear boundary at the adhesion site between the red and blue fibres (Masson’s trichome stain). (**d**) Optical microscopy of the La group shows a strong arterial adventitia contraction and both blue and red fibres in the laser-irradiated adventitia (Masson’s trichome stain).

The specimens that were subjected to the entire process comprised the Co + La group (*n* = 5), those that underwent fusion with the collagen sheet and Teflon sheathing without laser irradiation comprised the Co group (*n* = 5), and those that underwent Teflon sheathing and laser irradiation, but no fusion with the collagen sheet, comprised the La group (*n* = 5). The mean (standard deviation) maximum pressure resistance values for the Co + La, Co, and La groups were 303.6 (95% CI = 353.0–254.2), 50.8 (95% CI = 38.4–59.6), and 49.0 (95% CI = 21.3–80.3) mmHg, respectively, (Fig. 3a). The Co + La group exhibited a significantly higher resistance to pressure, with the wrapping maintaining its flexibility whilst it expanded under pressure. The indigo carmine solution leaked from the incision through the collagen sheet when a pressure of ≥250 mmHg was applied to three of the five samples. In the two remaining samples, sustained pressure of approximately 300 mmHg caused disconnections of the fixations of the arterial ligations, which allowed the indigo carmine dye to leak, but the collagen sheets remained intact. For the Co and La groups, the application of pressure led to gentle leaking of the indigo carmen dye from the gaps between the collagen sheets and the arterial walls (Movie 1).

#### Optical microscopic findings

The adventitia of the arterial walls was stained almost uniformly blue, whereas the mesh-like collagen sheet was stained light blue, white, pink, and red. At a low magnification, the arterial incisions in the Co + La group had caused the arterial adventitia to contract at both ends and become defective at the incision sites, whereas the arterial media, which consists of smooth muscle mainly, was stained red and closely contacted each side. At a higher magnification, the red fibres of the collagen sheet were mixed with the blue fibres of the adventitia where the collagen sheet adhered to the adventitia (Fig. 3b).

At a low magnification, the Co group showed contractions of the arterial adventitia, gaps at the sites of the arterial incisions, and the collagen sheet had peeled away from the adventitia. At a higher magnification, a clear boundary was evident between the red fibres of the collagen sheet and the blue fibres of the adventitia in the region where the collagen sheet adhered to the adventitia, and there was no intermingling of the fibres from the two sources (Fig. 3c).

At a low magnification, the La group showed strong contractions of and large gaps within the arterial adventitia, whereas at a higher magnification, the fibres within the laser-irradiated adventitia were red (Fig. 3d). The morphological observation of the second samples collected from different individuals indicated similar findings in each group (Co + La/Co/La group).

#### Scanning electron microscopic findings

**Figure 4:**
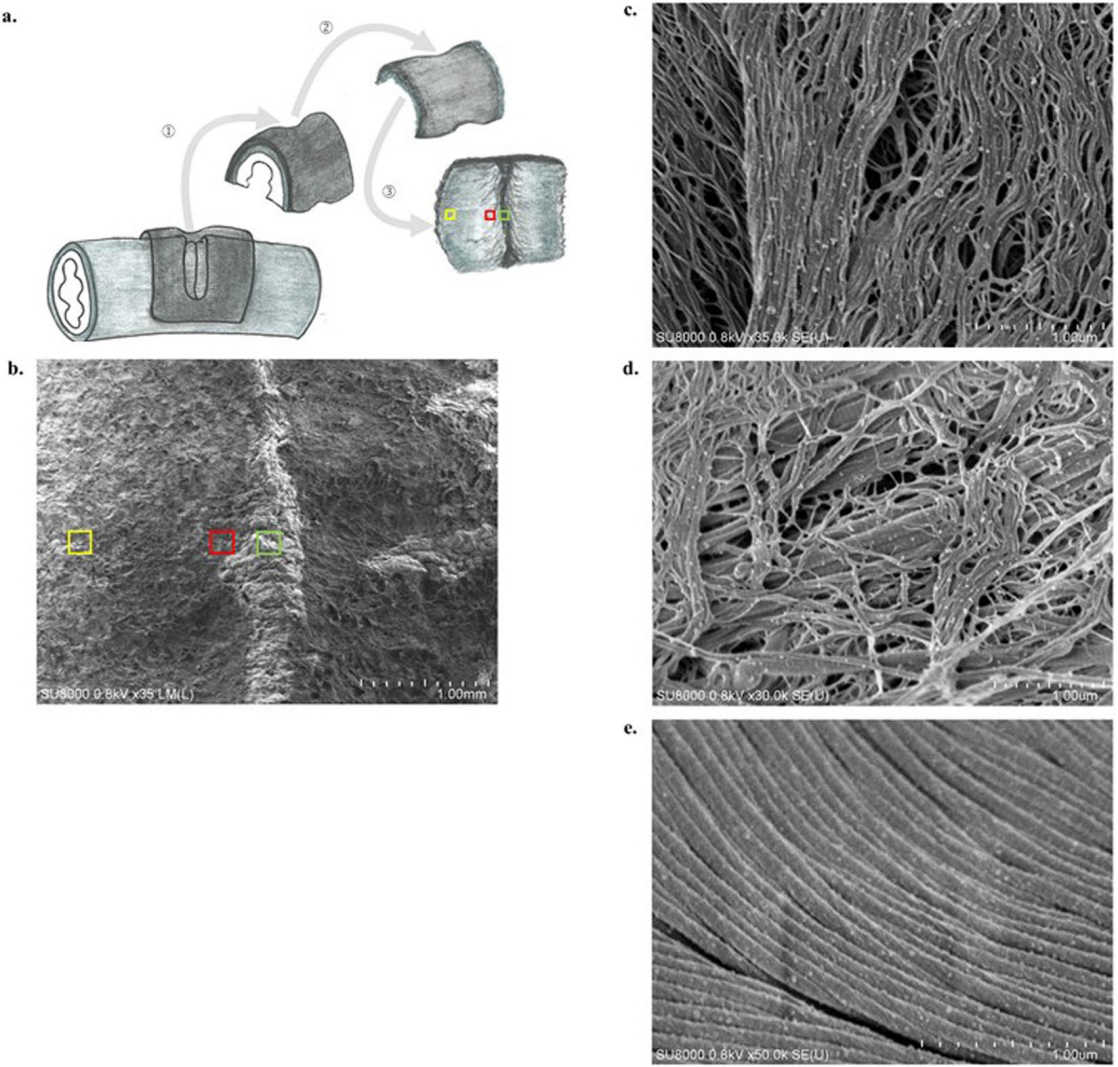
Specimen preparation of the group that underwent the entire laser tissue fusion procedure (Co + La group) for scanning electron microscopy and the findings. (**a**) Only the tissue with laser irradiation incision covered by the collagen sheet is extracted (① and ②), and processed specimen is examined from the luminal side in the incision site (green square), marginal site (red square), and outer edge (yellow square) (③). (**b**) A scanning electron micrograph (×35) of the bonding surface of the collagen sheet at three similar points described in. (**c**) At the central incision site, where the collagen sheet is in contact with the media, but not the adventitia, non-striped collagen fibrils are arranged in a dense sheet. None of the collagen fibrils have stripes that are a distinct feature of the biological collagen fibrils. (**d**) At the marginal site, two types of collagen fibrils with stripe and non-stripe appearance are present. Interdigitation between these different collagen fibrils from the collagen sheet and the biological artery is identified. (**e**) At the outer edge, only striped collagen fibrils from the biological artery are found.

To prepare for the scanning electron microscopic examination of the junction between the collagen sheet and arterial adventitia in the Co + La group, only the tissue that had been incised and fused by laser irradiation and covered with the collagen sheet was cut out (Fig. 4a-①). These specimens were soaked in 8% NaOH and repeatedly rinsed with distilled water to extract the collagen tissue only, thereby most of the arterial media with few collagen tissues melted and peeled off (Fig. 4a-②). These specimens were then examined from the lumen toward the adventitia at three points, namely, the incision site (green square), the marginal site (red square), and the outer edge (yellow square) (Fig. 4a-③). Scanning electron microscopy of the fused surface of the collagen sheet was performed at a low magnification (×35) (Fig. 4b**)**. Then, detailed examinations of the three sites specified in Fig. 4a were performed at a high magnification from ×35,000 to ×40,000. At the site of the central artery incision, the collagen sheet had collapsed into a gap formed by the contraction of the adventitia and it was in contact with the arterial media. Here, the collagen fibrils (mean diameter: 33.2 nm, 95% CI = 30.7–35.7 nm) that did not have a striped appearance were arranged in a dense sheet (Fig. 4c). No striped collagen fibrils were observed in this area. At the marginal site, interdigitation (interlocking entanglement) between two types of fibrils was observed, namely the unstriped collagen fibrils (mean diameter: 34.8 nm, 95% CI = 31.1–38.5 nm) from the collagen sheet and the striped collagen fibrils (mean diameter: 133.2 nm, 95% CI = 114.6–151.8 nm) from the arterial adventitia (Fig. 4d). In the outer edge, only striped collagen fibrils (mean diameter: 136.4 nm, 95% CI = 102.5–170.2 nm) from the arterial adventitia were present (Fig. 4e). Both samples had fibrils with a striped pattern and showed similar characteristic findings.

## Discussion

Tangled webs of the extremely delicate collagen fibres that make up the extracellular matrix maintain the morphology of multicellular organisms. In this investigation, we observed changes in the structure of biological collagen tissue caused by low-temperature heating, which caused reversible morphological changes to the collagen and did not denature other proteins. The bonds between the collagen fibrils loosened, and the collagen fibres swelled when the temperature was raised from 40 °C to 52 °C. We thought that if the swollen collagen fibres were bonded together under pressure, their loosened fibrils may interlock; thus, we incised the artery, ensured that it was in close contact to the vascular lumen in a longitudinal direction through the core to bond the cut surfaces, and then carried out laser heating. However, the arterial adventitia contracted from the incision, and only the arterial media was in close contact to the incision sites. Consequently, we attempted to fuse the arterial adventitia using a biomaterial-derived collagen sheet extracted from the bovine dermis as a bridge and applied the collagen sheet in a manner that was similar to the use of a haemostatic agent or artificial skin in current clinical practice. After applying low-temperature heating (46 °C) to the artificial non-crosslinked biomaterial collagen sheet, we observed fibril loosening and fibre swelling, which reflected those observed in the adventitia. We completely covered both sides of the adventitia with the biomaterial-derived collagen, which was centred at the site of the adventitial defect at the incision, closely in contact to the fibres, and then we heated the collagen using a laser without contact to a temperature range of 40–52 °C for approximately 5 s. Consequently, the collagen sheet and arterial adventitia fused together sufficiently firmly to enable the incision site to withstand high internal pressure. Examinations using optical and scanning electron microscopes revealed that the collagen fibrils in the arterial adventitia had interdigitated with those in the collagen sheet.

**Figure 5:**
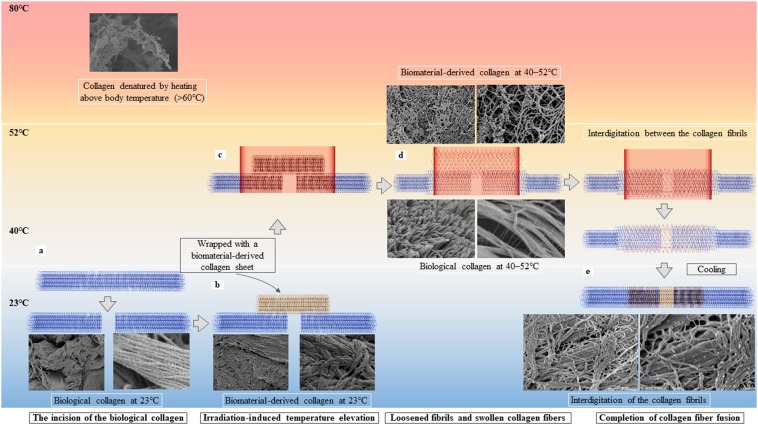
The principle underlying the laser tissue fusion method involves interdigitation between the biological and biomaterial-derived collagen as a consequence of heat-induced morphological changes. The interdigitation between the arterial adventitial (blue) and biomaterial-derived collagen (yellow) is illustrated in the context of temperature elevation. (**a**) When the cut surfaces at the incision site are brought close together, a gap forms in the biological collagen, (**b**) and the gap is wrapped with the biomaterial-derived collagen. (**c**) Laser irradiation of the biological collagen wrapped in biomaterial-derived collagen under pressure is performed, and heating at 40 °C–52 °C (**d**) loosens and swells the fibrils and fibres of the biological collagen and the biomaterial-derived collagen, and the collagen fibrils intertwine. (**e**) After cooling, the fibrils tighten, and the interdigitation of collagen fibrils is complete. When heated to >60 °C, the collagen fibres are irreversibly denatured and broken.

Figure 5 illustrates the interdigitation of the collagen fibrils based on heat-induced morphological changes. When an artery underwent a full-depth incision, the arterial adventitia contracted along the longitudinal axis, creating a gap (Fig. 5a). Wrapping the incision site with a biomaterial-derived collagen sheet enabled contact between the biomaterial-derived collagen and the arterial adventitia over a wide area (Fig. 5b). Heating the arterial adventitia that is wrapped with the biomaterial-derived collagen to 40 °C–52 °C (Fig. 5c) causes the fibrils and fibres from both sources to loosen and swell, which creates gaps in the fibril bundles and enables the fibrils of the adventitia and the biomaterial-derived collagen to intertwine where close contact is applied (Fig. 5d). As the temperature declines, the loosened and swollen fibrils and fibres return to their original bundled state, and the interdigitation of the two collagen fibril types is complete (Fig. 5e). When heated above 60 °C, the collagen fibres were irreversibly denatured.

Three conditions must be met to achieve robust tissue fusion. First, collagen fibrils and fibres that can be loosened and swollen by heating must be present. Second, sufficient heating and time are required to enable the collagen fibrils and fibres to undergo reversible morphological changes. Third, the collagen fibrils and fibres must be in close contact with each other over a sufficiently large area.

The smallest collagen tissue fibres are polypeptide chains (α chains) that link approximately 20 types of amino acids. Three α chains intertwine counterclockwise in a helical fashion forming tropocollagen, which is an elongated rod-like protein with a diameter of approximately 2 nm. Tropocollagen crosslinking between the chains forms collagen fibrils. In biological collagen fibrils, adjacent collagen molecules exhibit a striped pattern, because they are displaced from one another by 67 nm, which is about one-quarter of their length. The collagen fibrils converge further, forming bundles of powerful collagen fibres within the connective tissue^9–12^. Low-temperature heating using laser irradiation loosens the bundled collagen fibrils, thereby swelling the collagen fibres. The biomaterial-derived collagen sheet used in this study was fiberised collagen that is neutralised and lyophilised without crosslinking. It was reported that the occurrence of non-banded filaments required the solution to undergo a cold neutral step^23^. This is why it did not exhibit a striped pattern^23^. Nevertheless, like the biological collagen, low-temperature heating caused the bundled collagen fibrils in the collagen sheet to loosen and the fibres to swell. In an artificially crosslinked collagen sheet, in which the fibres that make up the collagen tissue are crosslinked randomly, the collagen fibrils do not loosen, and the collagen fibres do not swell. Indeed, we found no evidence of interdigitation in a pilot study involving a crosslinked biomaterial-derived collagen sheet.

Collagen tissue undergoes structural changes at temperatures that exceed body temperature^24^. A previous publication described structural changes in collagen tissues from a tendon under stepwise heating, and the measurements were based on Young’s modulus^24^. From 20 °C to 45 °C, collagen tissue loosening was observed. From 45 °C to 58 °C, Young’s modulus declined slowly, and the changes were no longer completely reversible by cooling. Above 58 °C, hardening of the collagen tissue was observed with Young’s modulus remaining stable or rising, and cooling did not reverse the changes. Consequently, the current study’s temperature ranged from 40 °C to 52 °C to induce the morphological changes necessary for tissue fusion, whilst avoiding irreversible changes.

The belief that one material can fuse with another via the penetration of extremely thin fibres into gaps over a certain surface area has been utilised to explain the adhesive strength of geckos’ feet as they climb up windows^25^. A gecko’s foot contains numerous fine fibre structures that resemble collagen fibrils. Even on a glass surface, which has only very fine irregularities, a strong adhesive force can be achieved by creating a large number of small Van der Waals forces over a large surface area^26^. Given that this Van der Waals force is inversely proportional to the sixth power of the intermolecular distance between the two interacting molecules, a strong bond is produced by ensuring close contact between the two materials to be fused^27^. The fused collagen fibres are intertwined due to the close contact between the collagen fibrils that were loosened and expanded by heating. After the fibrils cool again, there is also a mechanical bond that shrinks and bonds them to complete the interdigitation. Hence, the bonding force per unit area is more likely to be higher and more permanent than simple van der Waals forces^27^. Furthermore, although the close contact of the arterial adventitia could not be achieved as a consequence of the gap caused by contraction at the incision site, we thought that by affixing the biomaterial-derived collagen to form a bridge between the arterial adventitia, we could increase the contact area and obtain a greater bonding strength overall.

In addition to descriptions of its morphological changes, a variety of applications for collagen tissue, which also functions as a cytoskeleton, in wound healing have been reported. Collagen-based biomaterial can be used as a cell culture medium to provide a scaffold to aid cell growth^28^. Otolaryngologists use collagen-based biomaterial in this way for eardrum surgery, and techniques for repairing the tympanic membrane using a collagen sponge as a scaffold are advancing^29^. Collagen tissue, therefore, shows promise for promoting wound healing. Furthermore, for elderly people who produce and retain less collagen^30^, it may be possible to achieve more reliable tissue bonding in the intestinal tract and vascular anastomoses by strengthening these areas with additional collagen. Finally, we anticipate that our technique could be useful for rebuilding and repairing cartilage, dura, teeth, and other collagen-rich tissues, and that it could be applied to the nerves, the pancreas, and other organs in which tissue repair cannot be adequately achieved by suturing.

Hence, tissue fusion can be achieved *ex vivo* by heating collagen to low temperatures using lasers. Future work involving undertaking similar studies *in vivo* is necessary to investigate the durability of tissue fusions, to evaluate surrounding tissue reactions to tissue fusions, and to assess their impact on the tissue restoration process. This study’s findings have enabled us to elucidate the phenomenon of collagen fibre interdigitation that underpins the development of new tissue fusion technologies using contact-free heating that utilises laser irradiation. In addition to its use as a new minimally-invasive reconstruction technique in surgery, we hope that this technique can be applied to tissues that are difficult or impossible to repair or reconstruct using conventional techniques.

## Methods

### Heat-induced morphological changes to biological artery tissue and a collagen biomaterial sheet

#### Materials

Bovine carotid arteries (outer diameter 7 mm, inner diameter 3 mm) were harvested from Japanese Black cattle immediately after slaughter. A 100% non-crosslinked atelocollagen sheet with a thickness of 25 mm × 40 mm × 300 μm (Koken Co., Ltd., Tokyo, Japan) was used. This sheet was prepared by the following steps^31^. Atelocollagen was extracted from the bovine skin by protease treatment; this was followed by purification. The integrity of collagen and its triple helix structure was confirmed by SDS-PAGE and specific rotation. The atelocollagen solution was then lyophilised to make the atelocollagen sheet.

### Specimen preparation for optical microscopy

Two examinations were performed on arterial specimens collected from different individuals for each group and collagen sheets of different lot numbers for each group. After removing the connective tissue, an artery was cut into 5-mm long pieces. The collagen sheet was cut into pieces that were 10-mm long and 10-mm wide. For optical microscopy, the carotid artery and the collagen sheet pieces were washed in 10-mM phosphate buffered saline (PBS) for 1 min at 4 °C or 46 °C, fixed in 10% neutral buffered formalin at room temperature (23 °C) for 48 h, embedded in paraffin, sectioned at 5 µm, and stained using Masson’s trichrome stain (Supplementary Figure S1); Ad4 refers to the arterial adventitia washed at 4 °C, CS4 refers to the collagen sheet washed at 4 °C, Ad46 refers to the arterial adventitia washed at 46 °C, and CS46 refers to the collagen sheet washed at 46 °C.

Next, we scanned the slides with an Axio scan.Z1 (Carl Zeiss Microscopy GmbH, Jena, Germany) to create virtual slides. For each section of artery tissue, data describing the outer part of the external elastic lamina and the adventitia at six locations at 60° intervals within a 264 µm × 264 µm (pixel/µm^2^) area were extracted. Similarly, for each collagen sheet tissue section, data from six locations at approximately 1.5-mm intervals within a 484 µm × 484 µm (pixel/µm^2^) area were extracted. Using image analysis software (ImageJ, version 1.52b; National Institutes of Health, Bethesda, ML, USA), the relative proportions of the pixels associated with the collagen fibres that were stained blue or red were calculated, and the results from the specimens rinsed in PBS at 4 °C and 46 °C were compared using two samples in each group.

### Specimen preparation for scanning electron microscopy

Two examinations were performed on arterial specimens collected from different individuals for each group and collagen sheets of different lot numbers for each group. For scanning electron microscopy, the artery and collagen sheet specimens were immersed in 10 mM PBS for 1 min at 4 °C or 46 °C, fixed in 3% glutaraldehyde at 4 °C for 48 h, rinsed with PBS, placed in 1% tannic acid for 3 h with agitation, and rinsed in purified water. Then, the specimens were placed in 1% osmic acid for 3 h with agitation, rinsed with purified water, and they were placed for 20 min in solutions containing ascending concentrations of ethanol, namely, 70%, 80%, 90%, 95%, and 99% ethanol, and in anhydrous ethanol for 2 × 20 min. The specimens were shaken in a t-butyl alcohol solution that had been dehydrated for 1 day using a molecular sieve; this step was repeated, and the specimens were lyophilised. The specimens were then placed on a sample table (diameter: 15 mm), coated with osmium, stored in a desiccator, and examined using a Hitachi SU 8000 ultra-high-resolution field emission scanning electron microscope at Kyushu University’s Center for Advanced Instrumental Analysis. The diameters of the collagen fibrils that were processed at 4 °C and 46 °C were measured (*n* = 6) using ImageJ software, version 1.52b.

### Fusion of the biological artery tissue using biomaterial-derived collagen as a bridge

#### Materials

Tissues from the same bovine carotid arteries used in the microscopic analyses were used for the fusion experiments. The biomaterial-derived collagen sheet was used to cover the cut portion of the artery to form a bridge between the arterial adventitial layers. A 1,950 nm wavelength fibre laser system (SV-120 Lavertex; Suzuki Electric Industrial Co., Ltd., Japan) was used as the heating device. The irradiation time of the 1,950 nm wavelength fibre laser set with 10 mm irradiation diameter, 1.55 W average power, 5 Hz frequency, and 70% duty cycle was determined from the results of the following preliminary experiments performed before the laser collagen fusion experiments.: the artery was incised and covered with a collagen sheet in the same manner as in the collagen fusion experiment, and further covered with Teflon sheath, and a thermocouple was placed between the artery and collagen sheet. The temperature was measured 3 times at room temperature (23 °C). The temperature after 5, 7, and 10 seconds of irradiation was 40.4 °C (95% CI = 39.5 °C–41.3 °C), 46.2 °C (95% CI = 45.6 °C–46.8 °C), and 52.4 °C, respectively (95% CI = 51.9 °C–52.9 °C). The irradiation time was set to less than 10 seconds (9.9 seconds), where the duration of temporal change from 40 °C to 52 °C and from 46 °C to 52 °C was approximately 5 and 3 seconds, respectively.

#### Collagen fusion method

After the surrounding connective tissue had been removed under a stereomicroscope, the bovine carotid artery was cut into 4-cm long pieces. Using a surgical knife, a semi-circumferential full-thickness incision was made in the artery, and the cut ends were brought close together (Supplementary Figure S2a). The incision was then wrapped with a 300-µm thick collagen sheet measuring 13 mm × 10 mm (Supplementary Figure S2b). The entire specimen was then wrapped within Teflon film (15 mm × 10 mm), and the close contact between the collagen sheet and arterial adventitia was made without mixing with air using a stereomicroscope (Supplementary Figure S2c). Next, irradiation with preliminary laser settings was performed. The output-adjusted fibre laser was rotated at 30° increments to ensure that the irradiation remained central whilst it heated the arterial incision to a uniform temperature (Supplementary Figure S2d). After irradiation, the Teflon film was removed, and the specimen was cooled to 23 °C (Supplementary Figure S2e). The specimens that were subjected to the entire process comprised the Co + La group (*n* = 5), those that underwent fusion with the collagen sheet and Teflon sheathing but without laser irradiation comprised the Co group (*n* = 5), and those that underwent Teflon sheathing and laser irradiation but without fusion with the collagen sheet comprised the La group (*n* = 5) (Supplementary Figure S2).

#### Pressure resistance test

Supplementary Figure S3 summarises the pressure resistance test. A vinyl chloride infusion tube (outer diameter: 3.0 mm, inner diameter: 2.0 mm) was inserted into the lumen of the fused arterial stump and it was ligated and fixed with a 3–0 nylon thread. The arterial lumen was filled with indigo carmine solution (20 mg) (Daiichi Sankyo Co., Ltd., Tokyo, Japan), and the artery tip was clamped with vascular surgical forceps. The inserted tube was connected to a pressure gauge (Handy Manometer PG-100N-102R-H; Copal Electronics, Tokyo, Japan), and the indigo carmine solution was gradually injected into the tube using a 10-mL syringe, whilst the intravascular pressure was measured on the pressure gauge from the beginning of the injection until the indigo carmine solution leaked from either the incision or the ligation. The pressure resistance value was defined as the maximum pressure attained during pressurisation. The pressure resistance values and points of leakage were assessed by two investigators who watched videos of the pressure resistance tests with the arterial junction and pressure gauge displays showing on the same screen.

#### Optical microscopy

After cooling, the fused tissue specimens were fixed for 48 h in 10% neutral buffered formalin at 23 °C, and they were examined as described previously. Two samples for optical microscopic observation were prepared in each group (Co + La/Co/La group).

#### Scanning electron microscopy

To determine the specific forms of the collagen fibres at the junctions, the previously described NaOH maceration method^32,33^ was used with minor modifications. After cooling, the specimens were fixed in 3% glutaraldehyde at 4 °C for 48 h, rinsed with purified water, and immersed in an 8% NaOH aqueous solution at 23 °C for 4 days. The 8% NaOH aqueous solution was replaced daily. The specimens were rinsed in purified water at 23 °C for 4 days until all of the suspended particles had disappeared. The samples were then prepared in the same manner as described previously, and the surfaces of the junctions between the collagen sheets and the arterial adventitia were evaluated. Both samples for scanning electron microscopy were prepared in the Co + La group.

### Statistical analyses

JMP Pro software, version 12 (SAS Institute Inc., Cary, NC, USA) was used to evaluate the dispersion of the collagen fibres and compare the microscopy specimens regarding the proportions of the surface areas occupied by the collagen fibres, and to evaluate the dispersion amongst the Co + La, Co, and La groups in relation to the maximum pressure resistance. The results presented are the mean values from the total number of each sample type analysed. Student’s t-test of equal variance and Wilcoxon’s nonparametric test of unequal distribution were performed to assess the statistical significance of the results.

## Supplementary information

Supplementary figures and legends

Movie 1 (Resistance test Co+La 1)

Movie 1 (Resistance test Co+La 2)

Movie 1(Resistance test Co and La)

## Data Availability

All data generated or analysed during this study are included in this published article and its supplementary information files.
