# Supplementary material for: Principles and development of collagen-mediated tissue fusion induced by laser irradiation
Source: Sci Rep. 2019 Jun 28;9:9383. doi: 10.1038/s41598-019-45486-4 (PMC6598983; doi:10.1038/s41598-019-45486-4)
Supplement: Supplementary file 1 — Supplementary figures and legends [file 41598_2019_45486_MOESM1_ESM.pdf]

Article

Principles and development of collagen-mediated tissue fusion induced by laser irradiation

Shun Sasaki MD[1], Tetsuo Ikeda MD, PhD*[1,2], Shin-ichiro Okihara, PhD[3], Shotaro Nishimura, PhD[4], Ryu Nakadate[5], Hiroshi Saeki, MD, PhD[1], Eiji Oki, MD, PhD[1], Masaki Mori, MD, PhD, FACS[1], Makoto Hashizume, MD, PhD, FACS[5], Yoshihiko Maehara, MD, PhD, FACS[6]

[1]Department of Surgery and Science, Graduate School of Medical Sciences, Kyushu University, Fukuoka, Japan; [2]Endoscopy and Endoscopic Surgery, Fukuoka Dental College, Fukuoka, Japan; [3]Graduate School for the Creation of the New Photonics Industries and [4]Graduate School of Bioresource and Bioenvironmental Sciences, Faculty of Agriculture, Kyushu University; [5]Department of Advanced Medicine and Innovative Technology, Kyushu University Hospital, Kyushu University, Fukuoka, Japan; [6]Department of Surgery, Kyushu Central Hospital, Fukuoka, Japan

Authors' email addresses

Shun Sasaki: s_sasaki@surg2.med.kyushu-u.ac.jp

Tetsuo Ikeda: t-ikeda@surg2.med.kyushu-u.ac.jp

Shin-ichiro Okihara: s.okihara@gpi.ac.jp

Shotaro Nishimura: shotaro@agr.kyushu-u.ac.jp

Ryu Nakadate: nakadate@camiku.kyushu-u.ac.jp

Hiroshi Saeki: h-saeki@surg2.med.kyushu-u.ac.jp

Eiji Oki: okieiji@surg2.med.kyushu-u.ac.jp

Masaki Mori: m_mori@surg2.med.kyushu-u.ac.jp

Makoto Hashizume: mhashi@dem.med.kyushu-u.ac.jp

Yoshihiko Maehara: maehara@surg2.med.kyushu-u.ac.jp

*Correspondence to: Tetsuo Ikeda MD, PhD

Endoscopy and Endoscopic Surgery, Fukuoka Dental College, Fukuoka, Japan.

1-15-2 Tamura, Sawara-ku

Fukuoka 814-0193, Japan

Telephone and fax number: +81-92-801-0411

Email address: t-ikeda@surg2.med.kyushu-u.ac.jp

Supplementary Figure S1. Specimen preparation for morphological analyses. For optical microscopy, the carotid artery (5-mm length) and the collagen sheet (10 mm × 10 mm) specimens are immersed for 1 min in 10-mM phosphate buffered saline (PBS) at either 4°C or 46°C and fixed in 10% neutral buffered formalin at room temperature (23°C) for 48 h. For scanning electron microscopy, the specimens are similarly immersed for 1 min in 10-mM PBS at 4°C or 46°C and fixed in 3% glutaraldehyde at 4°C for 48 h.

Supplementary Figure S2. Laser tissue fusion method. **(a)** The artery is cut into 4-cm long pieces, a semi-circumferential full-thickness incision is made, and the cut ends are brought close together. **(b)** The cut portion is wrapped in a collagen sheet (13 mm × 10 mm × 0.3 mm). **(c)** The entire circumference is then wrapped in a Teflon film (15 mm × 10 mm), thereby completely covering the collagen sheet for uniform pressure bonding. **(d)** Irradiation is performed whilst rotating the 1,950-nm wavelength fibre laser (irradiation time 9.9 s; irradiation diameter 10 mm; output 1.55 W; frequency 5 Hz; duty cycle 70%) at 30° increments to heat the specimens to a set temperature. **(e)** After irradiation, the Teflon film is removed and the specimen is cooled to 23°C. Co+La (n = 5) represents the group that underwent steps (a)–(e), Co (n = 5) represents the group that underwent fusion with the collagen sheet and Teflon sheathing, but did not undergo laser irradiation, and La (n = 5) represents the group that underwent Teflon sheathing and laser irradiation only, but no fusion with the collagen sheet.

Supplementary Figure S3. Pressure resistance test. After the junction is formed with the artery, a vinyl chloride infusion tube filled with indigo carmine solution (outer diameter: 3.0 mm, inner diameter: 2.0 mm) is inserted into the arterial lumen, and the artery is ligated and fixed. The other end of the artery is clamped with surgical forceps. A digital pressure sensor (Handy manometer PG-100N-102R-H; Copal Electronics, Tokyo, Japan) and a 10-mL syringe containing indigo carmine solution are connected to the inserted tube. The liquid is injected manually, and the maximum resistance pressure is measured.

Fig. S1

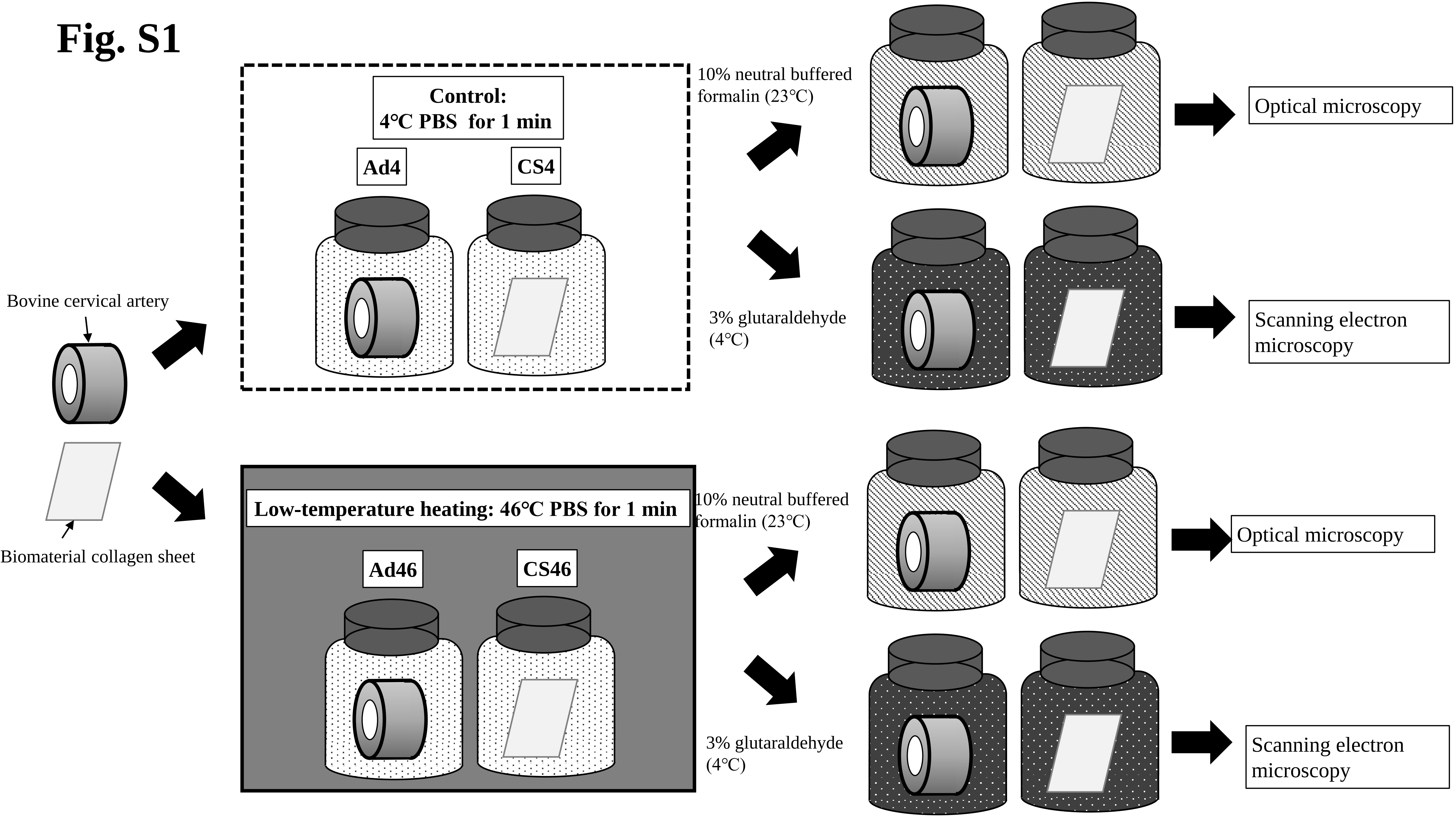
Control:
4°C PBS for 1 min
Ad4
CS4
Bovine cervical artery
Biomaterial collagen sheet
Low-temperature heating: 46°C PBS for 1 min
Ad46
CS46
10% neutral buffered formalin (23°C)
3% glutaraldehyde (4°C)
Optical microscopy
Scanning electron microscopy
10% neutral buffered formalin (23°C)
3% glutaraldehyde (4°C)
Optical microscopy
Scanning electron microscopy

Fig. S2

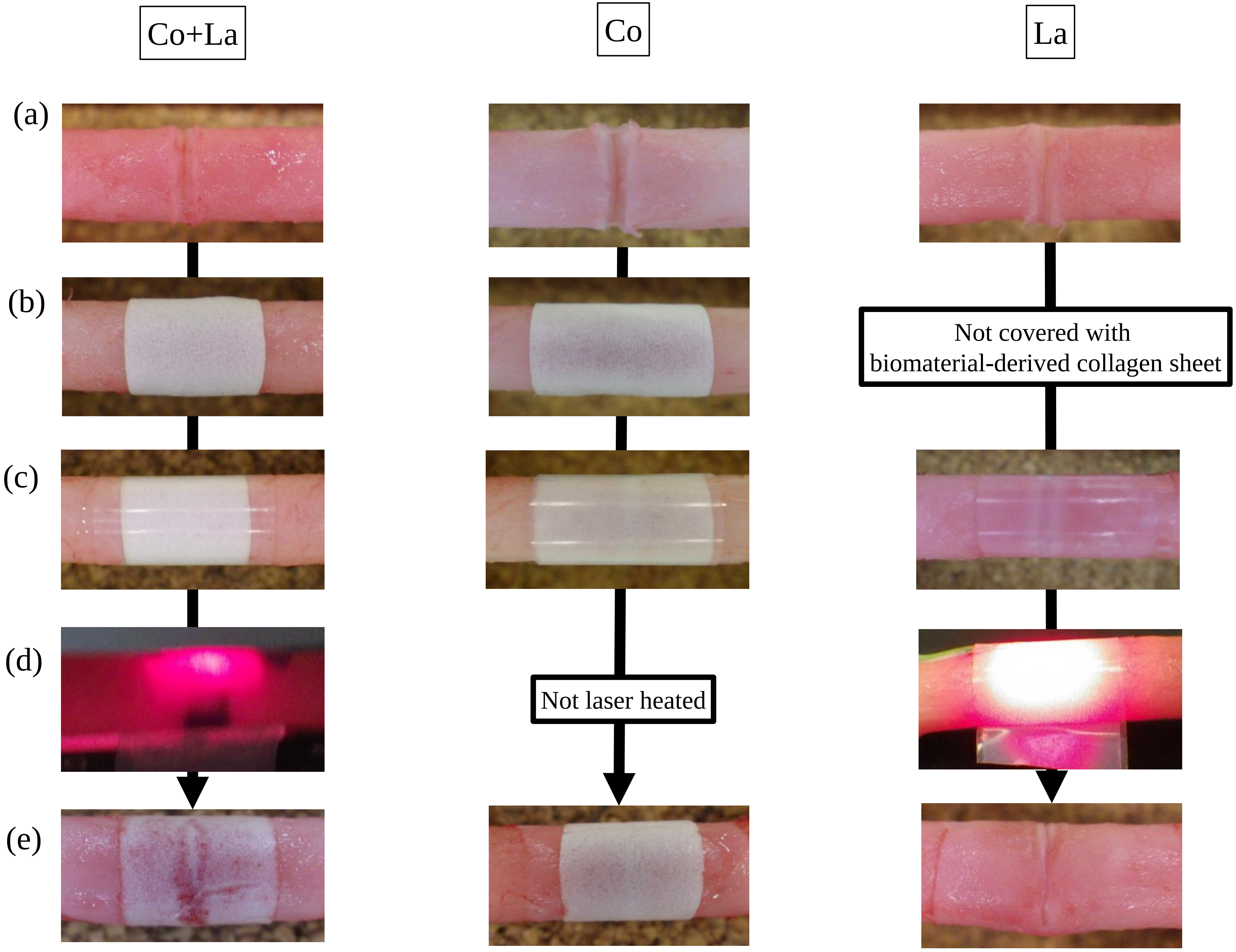
Co+La
Co
La
(a)
(b)
(c)
(d)
(e)
Not covered with
biomaterial-derived collagen sheet
Not laser heated

Fig. S3

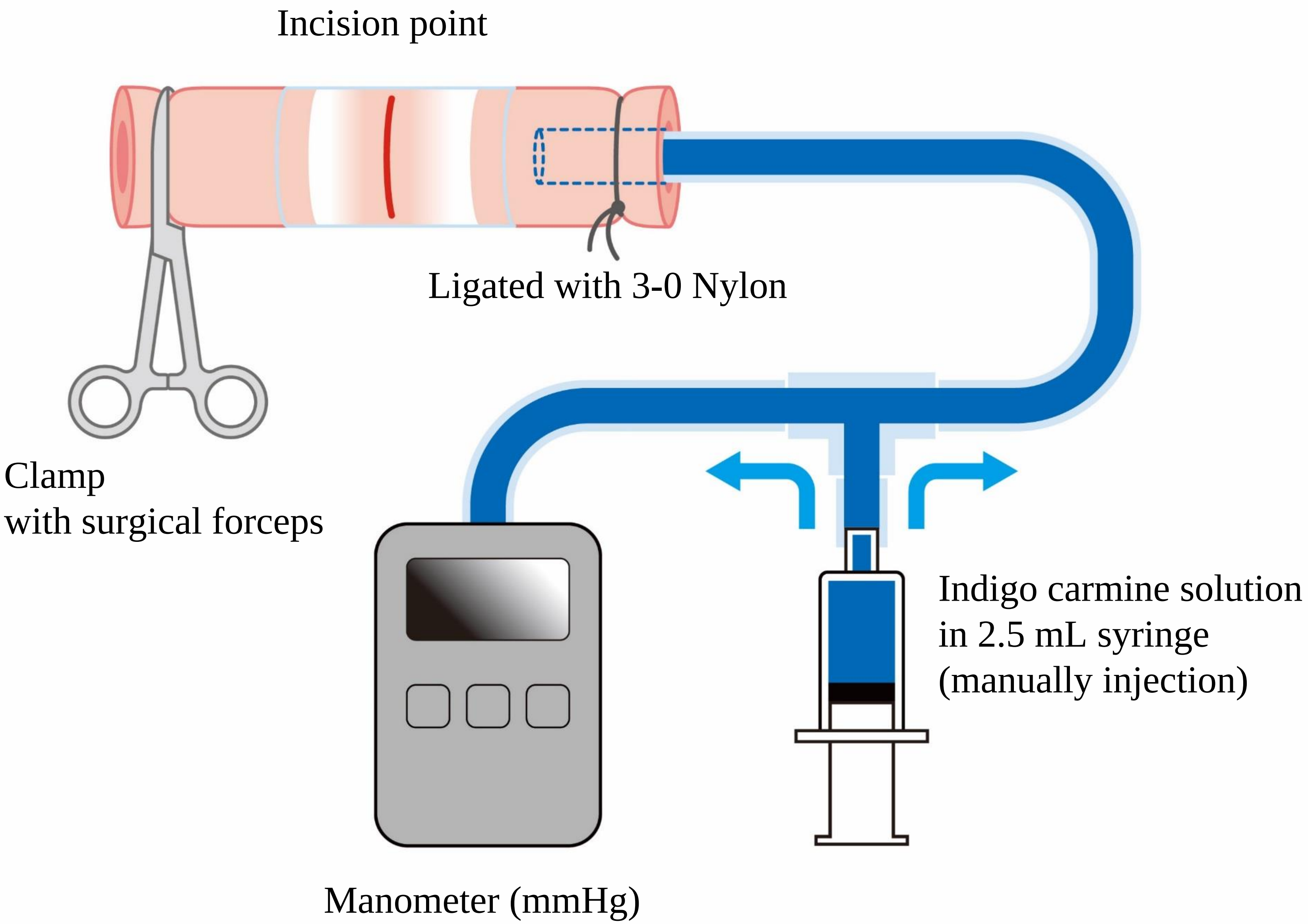
Incision point
Ligated with 3-0 Nylon
Clamp
with surgical forceps
Indigo carmine solution
in 2.5 mL syringe
(manually injection)
Manometer (mmHg)
